# Keeping Safe Together: Exploring Men and Women's Experiences of a Pilot “All of Family” Domestic Violence Intervention Program in Melbourne, Australia

**DOI:** 10.1177/10778012251366244

**Published:** 2025-08-20

**Authors:** Kristin Diemer, Anneliese Spiteri-Staines, Kate Hammond, Cathy Humphreys

**Affiliations:** 1 2281 University of Melbourne, Melbourne, Victoria, Australia

**Keywords:** domestic violence, family violence, “all of family” response, “whole of family” response, men's behavior change program

## Abstract

There are few interventions available to cater to domestic violence (DV) victim-survivors who are unwilling or unable to leave a violent situation. In recognition of this service gap, the Keeping Safe Together (KST) program piloted a support service to all family members in Victoria, Australia. This paper presents qualitative evaluation results for adult clients. Thirteen interviews were conducted with women victim-survivors (*n* = 8) and male perpetrators (*n* = 5). Findings revealed that women in the program experienced increased safety and supports while accountability processes were undertaken with men. Elements that required improvement included strengthening parents’ understanding of how DV impacts children, and an appropriate and safe family therapy approach to DV.

## Introduction

### Background

New programs are emerging to work with families where domestic violence (DV) is occurring, but victim-survivors are unable or unwilling to leave the violent situation. These families remain living together or are in regular contact, generally without a civil protection order in place. The models to support such families are in early development and are varied in their approaches. As such, the evidence base for what works is limited (Stanley & Humphreys, 2017). This article delineates issues emerging from the *Keeping Safe Together (KST)* program and discusses the challenges and opportunities of providing an “all of family” approach to DV as encountered by program clients. In *Keeping Safe Together*, the adult victim-survivors were women, while the perpetrators of domestic abuse were men. Gendered terminology throughout this article reflects this dominant pattern. We have also chosen to use the terminology of “all of family” rather than “whole of family” as consultation with the stakeholders in this area revealed that the latter term assumes the family is working towards being together as a “whole.” The former term more accurately represents an approach incorporating and providing services for all family members who are in contact with one another, regardless of whether or not a couple remains in a relationship.

### Service Gaps in DV Behavioral Change Interventions

Over the past several decades, conventional responses for intervening in cases of DV have been built upon the belief that separate services are required for men who use violence, and for child and adult victim-survivors (Pence & Paymar, 1993). Conjoint treatment of couples and families in DV situations has been considered unsafe, ineffective, and damaging to victim-survivors due to the likely premise that the abuse is a mutual problem to be solved (Goldner et al., 1990; Stith et al., 2011). This is partly why, in Australia, the presence of DV allows for a waiver of compulsory mediation or counseling in the Family Law arena (Commonwealth of Australia, 1975). Current and dominant intervention models are not designed for families that wish to stay together, or even where there is separation but substantial co-parenting or parallel parenting to be negotiated.

A number of issues exist with the separate and gendered model of intervention, particularly related to access for victim-survivors who remain in an abusive relationship, either due to a desire to stay, or due to factors outside of their control that prevent them from leaving. For example, women and children from migrant and refugee backgrounds often experience visa restrictions that tie their residency status to the perpetrator and limit their access to government supports and social safety nets (Humphreys & Campo, 2017). In Australia, Aboriginal women may not want to leave their cultural land and their extended family networks which are a central aspect of their identity (Meyer & Stambe, 2020; Murray, 2008). Affordable housing may be unavailable to DV victim-survivors due to a lack of supply, discrimination in the rental market, and unsuitability to cultural and/or religious needs (Meyer & Stambe, 2020; Murray et al., 2022). Women may have to make a choice between poverty or violence (Summers, 2022). Refuge accommodation may not be an option when it involves moving away from social networks and children's schools, prevents women from continuing their employment, or when they have accompanying male adolescent children (Murray et al., 2022). Separating from an abusive partner can leave women facing unemployment, homelessness, at risk of violence in alternative accommodation, and ultimately no safer (Diemer et al., 2017).

In addition, there is some evidence to suggest that removal from the abusive situation does not necessarily result in increased safety. In Australia, approximately 40% of DV incidents reported to the police are post-separation and often in the context of child contact (Humphreys et al., 2018; Humphreys & Campo, 2017). Many women have a genuine belief that it is better for themselves and their children to remain in an intact household, in spite of the abuse they experience (El Matrah et al., 2011; Stanley & Humphreys, 2017). The availability of services for intact families may support earlier help-seeking prior to the need for crisis intervention.

### Exploring “All of Family” Approaches to Intervention Programs

In light of the above issues, a case can be made for the need to explore different models of intervening with all family members where there is DV. Men's Behavior Change Programs (MBCP) and programs for fathers who use violence aim to facilitate women and children's safety by addressing the men's values, attitudes, and actions that drive abusive behavior (O’Connor et al., 2022; Scott et al., 2021). However, these programs can fall short on connecting and engaging with the women and children impacted by the violence (Day et al., 2019). The man using violence is the focus of a group intervention, though women and children are often named as the primary beneficiaries of this work. Yet the model of “partner” support work, or a women's service running parallel to the men's service has not necessarily been a successful model (Babcock et al., 2011). In the Australian context, MBCPs are often poorly funded and the women's support program can become marginal and sometimes perfunctory (Smith et al., 2014).

While some programs, such as the Caledonian program in Scotland, have worked towards more inclusive models (Ormston et al., 2016), the primary focus of MBCPs is for the work directly with men. Support work with women receives significantly less funding and as a result functions more as an “add on” to service provision. Contact with partners of the men in the programs is significant to assess women's safety, and as a form of accountability to monitor men's progress. While partner support can connect women and children to services as needed, it is not designed directly to meet the needs of women and children. This means that women and children's support is not central to MBCPs and often does not extend beyond the duration of their partner's attendance at the program (Smith et al., 2014). With high attrition rates across intervention programs targeting perpetrators of violence, including MBCPs (Cunha et al., 2022; Donovan & Griffiths, 2015), victim-survivors are at risk of losing the practitioner support that is dependent on their partners’ engagement in a program.

It has also been argued that intervention programs working with men without engaging or including women risk perpetrator collusion (Costello, 2006; Reimer, 2020). Sometimes, it isn’t until practitioners have spoken with partners that it becomes clear that treatment is not translating to improved behavior in the home, and that perpetrators are being deceptive throughout the duration of their treatment (Reimer, 2020). It is also the case that a primary motivation for men to attend an MBCP is the deterioration of their relationship and the possibility that his partner will leave him due to the violence (Fitz-Gibbon et al., 2024). In these situations, women may have given up on the relationship and it can therefore be difficult to engage them in the partner support to provide feedback about behavioral improvements.

While MBCPs are an important aspect of the landscape of DV interventions, there are varying degrees to which they achieve behavioral change with individual men (O’Connor et al., 2022). Additional or alternative options for addressing DV are therefore required. Some intervention programs to reduce men's violence have taken a couples-based approach, incorporating joint work with both the victim-survivor and the perpetrator (John et al., 2022; Méndez et al., 2014). Research into such programs has shown that some couples report positive changes for themselves and for their relationship during the course of the program, including increased relationship satisfaction, increased accountability from the perpetrator, decreased violence, and improved communication (John et al., 2022; Méndez et al., 2014). However, such programs are not appropriate for all couples, and are often designed to work with couples where the level of risk is low and the violence has not escalated (McConnell et al., 2020; Méndez et al., 2014).

Other programs go beyond couples work to also involve work with children. These programs utilize an “all of family” approach to DV intervention. Programs in the UK and Australia, such as the Doncaster Stronger Families and the Queensland-based Walking with Dads programs are examples of “all of family” interventions (Meyer et al., 2019; Stanley & Humphreys, 2017). These programs center on improving support offered to women and children, then draw in the work with men or perpetrators. For example, the Doncaster Stronger Families program reduced caseloads of child protection DV practitioners and redirected time to working with each family member. However, the program found that practitioners lacked confidence in working with fathers who used violence and consequently referred them on to specialist perpetrator programs, thereby siloing the work (Stanley & Humphreys, 2017).

The Walking with Dads program provides a more successful example of an “all of family” approach to DV intervention. This program involves training specialist child protection workers using the Safe & Together Model (Mandel, 2023) to work directly with all family members and conduct secondary consultations with child protection workers (Meyer et al., 2019). The model includes diverse strategies for keeping the perpetrator of violence in view, and proactive partnering with the non-offending parent (usually mothers). The work with men focuses on their fathering, understanding parenting behaviors, and recognizing the impact of their behavior on family functioning.

The development of “all of family” DV interventions are in the early stages. While some programs show positive signs of enabling work with all family members while maintaining safety for women and children, others demonstrate the need for further practitioner training and a wider understanding of this approach within the sector.

### Overview of the KST Program

The KST program was piloted to respond to the needs of families who required DV support but who chose not to or were unable to separate. KST was based in Victoria, Australia, and ran for a period of 18 months. The program was developed in collaboration between three separate services: one specialist women's DV response service, which led the pilot, a MBCP provider, and a family therapy center.

Family Coordinators, who worked with the women, and the program manager were employed by the specialist women's DV service. The men's workers, child worker, and administration assistant were employed by the MBCP provider. The family therapy center was included in the model in multiple roles: to provide clinical supervision, to assist in on-going co-design of the program through participatory action research, and to conduct regular staff practitioner meetings called co-production meetings.

Program practitioners were trained from varying theoretical frameworks and skillsets. The men's and women's workers came from DV backgrounds (i.e., case managers and men's behavioral change practitioners), while the child worker and family therapists/family coordinators came from therapeutic backgrounds. The two external clinical supervisors from the family therapy center also came with a therapeutic lens, one of whom had experience working with families recovering from DV.

The program staff across the three services were co-located within the specialist women's DV service on KST days. All practitioners shared an open-plan office to encourage informal case management discussions. Formal case management meetings in relation to particular families were arranged on an as-is-needed basis.

Separate services were provided to the mothers, fathers, and the children with co-case management by the practitioners. These individual sessions involved first, information gathering to allow practitioners to understand the client's situation, before moving on to psychoeducation and developing DV literacy. This stage asks men and women to reflect on their behavior with the goal of alleviating self-blame for the women and increasing accountability among the men. In addition to in-person sessions, clients were able to access unscheduled support as needed over the phone. This included calls from workers to check in, emotional support, and safety planning.

Family sessions or joint sessions between the parents were only offered after individual work had been completed, and only if deemed appropriate and safe to do so. Each person received individual counseling services, DV case management support, and referrals to other services as appropriate, including MBCP, Caring Dads, and alcohol and other drug (AOD) services. The design of the KST program was such that all family members agreed to participate and were aware that the other family members were receiving support for DV.

The majority of the families were referred into KST from Child Protection (36%) or from child and family services and/or other internal referrals (36%). Five women and six men were recorded as self-referrals. Staff indicated that some clients had heard about the program from court, Family Services Victoria, Caring Dads, and other MBCPs.

While the program was voluntary, there were often strong formal motivators to attend (child protection stating that retaining parenting rights was contingent upon attendance) or informal motivators (partners “threatening” to leave). The program was originally designed for families where the level of violence was deemed low to medium risk. However, based on a standardized risk assessment, most referrals into the program involved medium-high risk families. The KST program design was subsequently modified to accommodate higher risk families by including increased safety planning, and ensuring appropriate risk mitigation strategies were implemented when considering joint sessions including both partners.

The program was designed to be flexible to suit individual client and family needs, and to allow for cultural adaptation. The majority of clients being seen at KST were from Culturally and Linguistically Diverse (CALD) backgrounds—a population that often faces additional barriers to support. Some of the KST staff had specialist training and expertise in working with CALD communities and shared this knowledge with other staff through in-house training for the team. This was to ensure that staff were sensitive to cultural issues and norms. KST also had a small number of Aboriginal clients engaged and offered additional support by an Aboriginal Community Controlled Organization (ACCO).

There was no limit on number or frequency of sessions for participants while the program was running. At the time of this study, “completion” of the program was only recorded for a small number of clients who had withdrawn. Unless withdrawn, KST clients remain “active” so that they can return to the program on an as-needed basis. Of the two men, three women, and one child who “completed” the program (i.e. formally withdrew), the engagement periods were between 9 and 18 months. Overall, the fact that most client files remained “open” and the relatively long length of participation measured for cases indicates the need for long-term work.

The KST pilot was funded by the Victorian Government for a period of 18 months. At the end of the funding period, the delivery organizations committed to self-funding the program for a period of three months while further funding was sought. The program was unable to secure additional funding beyond this point and the program consequently ended.

## Methodology

In recognition of the need for further evidence on the effectiveness and appropriateness of “all of family” approaches to DV interventions, this program evaluation sought to understand the following question: *How have adult clients experienced the KST program and what, if any, changes to their safety and circumstances have occurred?*

This paper utilizes qualitative interview data gathered through a larger evaluation of the KST program. This evaluation took a realist evaluation approach and centers not only on intervention outcomes but also how they are produced, and what is significant about the varying conditions in which the interventions take place (Pawson & Tilley, 1997). The overall evaluation explored the contextual conditions that make the KST intervention effective / not effective and searched for lessons about ways in which both the context and program design produce outcomes.

The evaluation incorporated both formative and summative elements using a mixed-methods approach including outcome-related data in client files, semi-structured interviews, focus groups, and two case studies which demonstrated how the KST program worked. Data was collected between March-June 2019 in two parts. Part one focused on clarifying the program model and evaluating the implementation of the KST program. Part two focused on outcomes for individual family members including adult women, men, and children/ young people who were involved in the KST program. This evaluation was commissioned in the final six months of the program and therefore was unable to collect baseline (pre-program) interviews from clients.

To understand the impact of the program, KST clients were introduced to the evaluation through their case worker. The research team then contacted potential participants and undertook confidential recruitment. Thirteen semi-structured interviews were conducted with adult clients. Participants were provided with a Plain Language Statement, which set out the process for the interview and ensured that participants were providing informed consent. Due to the sensitive nature of the discussion, participants were made aware that all interview questions were optional and they were able to take breaks, or end the interview if needed.

Interviews took place from a feminist social constructivist perspective, emphasizing the place of language in the construction of social reality. Participants were encouraged to speak about their lived experiences (Sarantakos, 2005) associated with the evaluation. Conduct of the interviews drew on a relational empowerment methodology to create a caring and communicative space which enhanced individuals’ abilities to clarify their feelings and thoughts (VanderPlaat, 1998). This approach enabled the collection of rich data in a safe environment for participants. Interviews were conducted in-person by three researchers. Participants chose the time and location of the interview as convenient to them, with most taking place at the KST office around their service appointments.

The researchers conducting the interviews were highly experienced in research with victim-survivors and perpetrators of DV. Participants were supported to feel safe to speak about their involvement in the programs and their experience of, or use of violence. Interviews with victim-survivors can carry risks, including the potential for distress, discomfort, and further violence as a retaliation for participating (Elsberg & Heise, 2002; Hamberger et al., 2020). Interviewing perpetrators of DV could likewise trigger strong emotions including distress and shame. Further, there may be a risk that the interviewer adopts collusive or justifying behaviors (Reimer, 2020). The experience of the researchers was therefore important to ensure that these risks were appropriately managed. It is also important to remember that many victim-survivors of violence find participation in research to be cathartic and empowering, even when it is associated with the release of strong emotions during the process (Valpied et al., 2014).

Interview transcripts were de-identified and participants were assigned a pseudonym to protect their privacy. The data were coded using the steps outlined by Braun and Clarke (2013) to determine common themes and overarching ideas. Two researchers coded the same three interviews, after which a comparison took place and a coding framework developed to ensure consistency in analysis. The remaining interviews were coded according to this framework.

Ethical clearance was obtained through the University of Melbourne ethics committee [ethics ID 1853008.1]. Safety protocols were in place to address the issue of confidentiality and disclosure of any current, unreported child abuse, or risk of self-harm.

Final interviews included eight (36%) of the 22 women participating in the program at the time of the evaluation and five (22%) of the 22 men.

Analysis of the interviews with children / young people has been published in a separate Brief Report (Diemer et al., 2024). This paper is limited to the results of the individual qualitative interviews with mothers and fathers.

## Results

### Interviews With Women

Of the eight women interviewed, six were still in a relationship with their partners. Women's ages ranged between 32 and 51, and most were Australian born with two identifying as being from culturally and linguistically diverse (CALD) communities. Half of the women's children were also attending the KST program. Four of the men interviewed were partners to the women interviewed (Table 1).

**Table 1.** Contextual Issues for the Women Interviewed (*n* = 8).

Issue	count
Number of women no longer in relationships with men also in the KST program	2
Number of interviews conducted with matched ex/partners of the women included in the evaluation	4
Number of women who separated from their ex/partner during the program	2
Number of women with a civil protection order taken out against ex/partner	1
Number of women with a civil protection order separating ex/partner from their children	1

#### Overall: Filling a Service Gap

Women were very positive about the program and expressed a preference to attend on an on-going basis. Some had not realized that they were living with DV when they first became clients, and those who suspected DV felt validated by what they learnt through KST. Equipped with the tools to seek help and support, women were able to make informed decisions whether to separate from their ex/partners, or use the KST program support to work towards a safer family environment. For example, Maria and her husband were referred to KST through child protection. They were told that if they wanted to stay together in a relationship, and keep their children in their care, then attending KST was their only option.

Chris and Maddy were introduced to KST at the magistrate's court when seeking a civil protection order following a DV incident. Similarly, Sima found the program after she and her husband attend court and her husband was served with a civil protection order in response to his DV. Sima asked to vary the order to allow for her husband to live with her, under the condition that he would not hurt her. Since both Maddy and Sima continued to live with their abusive partners, they would have found the support from a specialist DV service highly constrained or not available. As Maddy described, “we’d probably be in court and separated by now without it [KST]”.

For some women, the name of the program and knowing that it was aimed at supporting families living with DV was what initially attracted them to participate. Rana was drawn to KST as the materials mentioned couples “remaining together” and she did not want to leave her partner.

To help us as a family be healthy…program to help with communication and issues. (Rana)

Nobody wants to break their relationship and that's how I came into this program, with the help of my GP. ‘Keeping safe together’ is an important name because every wife wants to be orientated with the husband. (Gina)

#### Emergent Theme: Empowerment

Increased empowerment was an immediately noticeable theme from the women's interviews. They greatly appreciated being provided support and assistance to identify and name behavior from their ex/partner as either appropriate or inappropriate. Their involvement with KST also gave them tools to communicate with their ex/partner and other people about what they were experiencing. The psychoeducation component of the KST program was identified as particularly helpful to enable DV literacy and make informed decisions about their lives.

[I can now] honor my own experiences rather than forgive things that are unforgivable. Reclaim control and power using language. (Rachel)

[My worker] helped me feel comfortable to tell him [ex/partner] my problem. (Sima)

[I] stopped blaming myself for what he is doing. (Chris)

Women also appreciated having the practitioner as an impartial third party to speak to. Most women had not felt comfortable talking to their friends, especially prior to their involvement with KST. Some reported feeling more comfortable confiding in family and friends as a result of KST. These participants felt their experience had been validated and KST participation helped them develop a wider social support system.

KST… is good to have a non-biased person offer their view. (Chris)

I got a lot of validation and reinforcement. I had felt quite isolated before. I didn’t talk to my family about it. I received validation [through KST]. (Rachel)

#### Emergent Theme: Improved Family Relationships

Women reported improvements in their family relationships including with their own children, their ex/partner, and in some instances also reported improvements in the father's relationships with their children. Women attributed these improvements to KST's focus on developing effective and respectful communication skills.

I have changed my life so much. First the recognition that you can’t change anyone by arguing, pushing—the person needs to have their own choices. (Maria)

He has changed a lot since KST, our relationship has grown stronger, and he has learned to respect everyone the same (Sima).

Some mothers expressed concern that their children were witnessing unhealthy relationship behaviors before KST. Being in the program helped them learn how to communicate better with their children and support them to understand what a healthy relationship looks like. In some cases, this resulted in more trust and openness in the home.

[Showing the children that] life doesn’t have to be yelling and screaming. You can change yourself; you can talk. (Rana)

With this program I started to be alert for my safety, before I never realized how important my safety is, for my kids as well. (Gina)

For example, they [the children] would previously hide everything on their phones from me. Now they will approach me and ask what to do if they get contacted by an unknown number and show me what they are doing. (Maddy)

#### Emergent Theme: Identifying Abusive Behavior and Safety to Speak-out

Women who had previously felt unsafe around their ex/partner reported increased feelings of safety while involved in KST. While some of this was a result of men changing their behavior, more often it centered around women becoming more adept at identifying manipulative and dangerous behavior, or having a safety plan in place.

While all women were better able to recognize inappropriate and unsafe behavior, some also reported that they felt new confidence to inform their ex/partner when their behavior was abusive. This confidence was due to having KST support for both parties.

He [partner] used to say that it was all in my head that his behavior was acceptable. But now other people are saying it is not ok. Them listening and saying you’re not going crazy, it's a relief it isn’t you [your fault]. I feel safer to call out the bad behavior and turn to services for help when I feel unsafe. (Rana)

[I] feel more able to tell [him] what is acceptable behavior…Also prepared to leave him and knowing this has impacted on his behavior. (Chris)

With this program I started to be alert for my safety, before I never realized how important my safety is, for my kids as well. (Gina)

One woman attributed changes she saw in her ex/partner not just to communicating more about his behavior, but also the approach KST took during sessions with the men. Specifically, that the men were given space to tell their stories and felt heard:

If there had been blaming, [he would have] a knee jerk reaction, rather than allow a space for him to be educated. [He] had not realized he was violent, and he was supported in learning about his behavior without finger pointing.” (Rachel)

Unfortunately, feelings of improved safety weren’t always maintained. Some women reported that though they initially felt safer around the ex/partner when first becoming involved in KST, they believed the change was temporary. One participant stated that progress “wore off over time. Only so much new learning before old habits kick in” (Rachel).

Some program participants also attributed an increased feeling of safety to leaving the relationship rather than working on behavior change alongside their ex/partner. The sessions with KST had provided space and support for them to assess what was best and safest for them personally, and how they could realize this safety. For those who chose to leave their relationship, they were appreciative of the support that KST had provided towards them doing so, including practical support such as financial and housing assistance.

#### Emergent Theme: Awareness of Children's Needs

Women attended the KST program primarily to reduce their ex/partner's use of violence and to support relationship repair. While their children's needs and the impact of the violence was in the front of mind for many, some required prompting to draw focus towards how and whether KST had impacted their children's wellbeing. One exception was the conversation with Maddy. She said she had never felt unsafe with her partner, however she had been concerned for the safety of her eldest child.

He [father] knows that he can’t do what he's done to her [daughter]. He tries not to deal with her, [gives her] more space. He used to yell, constantly repeating. He was loud and that would set her off. He knows when to stop now. (Maddy)

For most women, a safe home environment for their children, and improvement in parenting practices, was viewed as a secondary objective of the program. Yet, when asked, they were able to describe overarching changes in their children during the KST program, but did not expand in great detail. They instead primarily saw the KST program as a conduit to improving their relationshipd with their children. Improvements for their children were secondary or incidental.

### Interviews With Men

Five men were interviewed in total. They had been involved in the KST program for between 3 and 6 months. At the time of interview, four of the men were still with their partners and one had his relationship end during the program. Two of the men had civil protection orders against them. Their ages ranged from 29 to 56 years (Table 2).

**Table 2.** Contextual Issues for the Men Interviewed (*n* = 5).

Issue	Count
Number of interviews conducted with matched ex/partners of the men included in the evaluation	4
Number of men separated from their ex/partner during the program	1
Number of men with a civil protection order taken out against them	2
Number of men with a civil protection order separating them from their children	1

#### Emergent Theme: Acknowledgement and Reflection

Accounts provided by the men were more varied compared to those from the women. Four of the men had started to become more reflective as a result of their involvement with the program, and acknowledged that they had issues to work through. Prior to KST, some men had not realized that verbal abuse was a form of DV, and the program supported them to understand that their past behavior needed to be addressed. They developed a greater understanding of how their actions could make their ex/partners feel.

I have learnt through the program that yelling and swearing is a form of DV. Didn’t know this before. … [I] need to take responsibility for my actions—no matter how much they [ex/partner] drive me nuts. (Sam)

The men were grateful for the counseling and psychoeducation they received, which helped them to change their outlook and perspective on their use of violence. Through this they were able to recognize their role in creating a safe home environment.

I like it [the KST approach]. We all see things differently. But the essence [of the program] is creating an environment where everyone feels safe.” (Jalal)

Straight away I started realizing my behavior had not been respectful. This was due to differences in culture and the financial pressures of living in Australia. I understand now that my Italian/Spanish background is loud, and I don’t communicate disagreements well. (Marco)

Some men however, had not yet benefited from psychoeducation, and at the time of interview remained resistant to taking responsibility. The men who were at early stages in the program had made some progress but continued to blame their (ex) partners for “driving” them towards abusive behaviors. Some men also articulated that they were frustrated with their ex/partners and believed themselves to be doing all the “hard” work of behavioral change.

The IO [intervention order] gave her protection. That gave her protection from me in a lawful way, but also gave her permission to push my buttons. (Sam)

KST has brainwashed my wife. From the beginning she started talking about her rights, financial and otherwise. The relationship has become more strained as a result of all the information KST filled her head with. (Amin)

While some men had not yet developed any insight into their own behavior, all planned to continue attending KST as they had begun seeing improvement in their relationships. However, some men reported the improvements were related to an improvement in *her* behavior.

In the past two weeks her behavior has improved. I asked [case manager] to pass information on to [her case manager]. I told them to get her to look at her own behavior. I did not want to hit her [his wife] but she pushed me too far. I think they understand so I will keep coming [to KST]. (Amin)

#### Emergent Theme: Relationship Repair

The main expectation of men going into the KST program was that it would be a form of relationship counseling. The focus on remaining together in the relationship had greater appeal for the men as compared with women. A few men expressed a desire to do more couples work in the program. However, their description of the work they wished to do in joint sessions indicated they lacked understanding of their role in creating an unsafe environment for their partner. Sam expressed disappointment that he and his ex/partner had not made it to the “couple's counseling” stage, and he attributes this to why they may have split up. Amin was resentful that they couldn’t have joint sessions, stating that “[t]heir first mistake was to separate me and my wife. We came there together. That shows our commitment” (Amin).

Nevertheless, the majority of men did appreciate individual sessions. This meant they could focus on their own work with a counsellor before any joint work. Some men said that having their ex/partner involved made them feel like they were working together—even if they were having individual sessions—because they were both showing that they were committed to repairing the relationship. This belief that their ex/partner had an equal responsibility to work on the relationship reflects a lack of insight into their role in the abuse.

[My wife] doing the program as well shows she is committed to the work as well…Teamwork, not only I’m the bad guy. (Marco)

I haven’t been able to see my kids because of the IVO [civil protection order]. But I never did anything bad around the kids. It's a different story for [partner]. She's in the program too [KST] but gets to see them [kids]. (Joshua)

Other men were better able to recognize their role in repairing the relationship, and the impact their behavior could have through the program. The word “respect” was mentioned by three of the five men interviewed.

Rather than trigger negative emotions, respect each other a bit more and be more reflective. (Jalal)

#### Emergent Theme: Behavior Management

Even though the men interviewed were not willing to take full responsibility for their actions, most recognized that their behavior could be improved. Only one man among those interviewed blamed his ex/partner entirely for his behavior. Even when in denial about how much they were responsible for, men were generally able to recognize the need to improve their behavior and better manage their emotions.

Good [stuff] comes out of the program. If not for that, I would have breached the [protection] order…Learned some [stuff] out of it.” (Joshua)

Less concerned about my position in conversation and to think about the other person's position…More empathetic to other person's emotions, rather than being right.” (Jalal)

As a result of the KST program some men described coping mechanisms they were now able to reach for in situations when they may have previously used violence.

Learned to walk away in more situations, rather than smash stuff like I used to. (Joshua)

[Worker] had showed me the stages of escalation of disagreement, and taught me to stop before conflict escalates, and to be aware of the consequences. (Marco)

One man, who had separated from his ex/partner, was still committed to attending KST because of the effect it is having on his coping skills. He reflected that his continued attendance will “benefit myself to stay involved and will benefit others I have disputes with” and that he has “new skills and coping mechanisms in future” (Sam).

Despite learning how to better manage situations generally, men still struggled with consistency in different settings, especially in the context of AOD use. As one participant shared, “I’ve learnt new techniques to diffuse the situation, but when alcohol is involved, I can’t seem to get the hang of it” (Sam).

#### Emergent Theme: Supportive of the Program

All men had positive feedback about their KST practitioners. They felt comfortable speaking with the case managers and in general felt “heard”. This was important to their on-going participation, even in cases where they were no longer with their partners. Some reported they were hesitant at the beginning, expecting that the program would be “against them”. This discomfort was soon dispelled by the practitioners who worked with them, rather than against them.

Lots of incentives to share thoughts…Not being criticized. Makes you feel comfortable. Someone listening, someone cares. (Marco)

It will benefit myself to stay involved and will benefit others I have disputes with. New skills and coping mechanisms in future. (Sam)

I’d be feeling fairly desperate without it, I’d be scratching my head. Probably do counselling but not 100% comfortable to [do that]. (Jalal)

One participant was especially grateful for the one-to-one sessions. He had been attending another program focused on men who use violence and are fathers (Caring Dads) but felt unable to speak up during group sessions. One-to-one sessions in KST provided a safe environment for him to voice his feelings and thoughts, whereas previously, he would “Bottle [things] up until breaking point” (Joshua).

#### Emergent Theme: Parenting

As was the case with most mothers, the fathers did not often speak about the impact the program had had on their children or their own parenting practice. Some reflected awareness of problematic behavior toward their children but were unable to enact appropriate alternatives. They consistently mentioned learning a safe practice, which was to avoid confrontation.

I seem to be doing nothing right. Better off doing nothing [not disciplining] than doing things wrong. (Jalal)

Many of the fathers were focused on the program's impact on their ex/partner's parenting.

She [now] takes time to explain to them why a certain behavior is bad, rather than just telling them not to do things. (Jalal)

Overall, men were more focused on themselves and their relationships with their ex/partner, and rarely referred to their children. Comparatively, women were more acutely aware of their children and the impacts of the violence on them.

## Discussion

The KST program was an innovative pilot that filled an important support gap for families experiencing DV who did not wish to, or were unable to separate. The findings from our evaluation demonstrated that for this group of participants the overall results were positive, although reinforced the need for extended support to facilitate long-term safety and behavior change.

Women reported experiencing increased safety as a result of the program. In some cases, increased safety was attributed to men changing their behavior, while in others, it was a result of women's increased DV literacy and safety planning. Women felt validated in their experiences; gained increased understanding of healthy relationships, and were better informed to make decisions about their relationships. Women also developed improved communication with their children.

However, few women and men saw the KST program as primarily a conduit to improve the lives of their children. In their eyes, this was a secondary, or incidental benefit of the program. The men in particular focused on themselves and their adult relationships, rarely referring to their children and having little awareness of whether their engagement with KST had impacted their children. Past research consistently shows that violent men often fail to recognize the impact that their use of partner violence has on their children (Alderson et al., 2013; Harne, 2005, 2011; O’Connor et al., 2022), and this tendency appears to be present among KST clients. Future programs that utilize an “all of family” approach may benefit by giving greater focus to supporting parenting capacity and centering children's wellbeing in their behavioral change goals. One strategy would be for fathers in the program to meet directly with the child practitioner, without the child present, and if safe to do so. Fathers may be better able to understand the impact of their violence on children if hearing from someone who is working directly with their child(ren). There may be some additional learnings to take from Queensland's Walking with Dads program, which also adopted an “all of family” approach and centered the program around supporting men to recognize and change harmful behaviors in the context of parenting (Meyer et al., 2019).

Mothers would likewise benefit from more focused elements related to parenting and the impacts of DV on children. However, interviews suggested that it was difficult to focus on the children's narrative when the situation with the parents can be so demanding of attention. As the father's use of violence is the cause of the violence and disruption in the household, mothers may prioritize these issues. The mother-child relationship, which is a key protective factor for young children, is often directly attacked and undermined by perpetrators (Kertesz et al., 2021). In addition to the significant impacts this can have on both the mother and child's mental and physical health and self-worth, it can also interfere with attachment processes (Katz, 2019). It is possible that mothers in the KST program had experienced the undermining and targeting of their relationships with their children, which may be impacting their capacity and confidence in their roles as mothers. Like fathers, mothers may also benefit from meeting with the child practitioner, as well as more individual sessions that focus solely on the mother-child relationship and parenting challenges. Communicating to mothers the impact of DV on their children, and re-focusing their goals within the program towards children's safety and wellbeing may be more fruitful due to their being the targets rather than the perpetrators of violence. When working with mothers, the added challenge is communicating the impact of the violence on children without implying that mothers are at fault or that their parenting capacities are coming under scrutiny, thereby replicating a “failure to protect” discourse (Buchanan & Moulding, 2021; Nixon et al., 2017).

Emphasizing the child-centered nature of the program is paramount due to the severe and potentially lifelong impacts of growing up in a violent household. In the immediate term, the trauma of the abuse may be impacting multiple domains of functioning; driving or exacerbating various mental health disorders, developmental concerns, and impeded biological process (Asiedu & Baliki, 2025; Margolin & Vickerman, 2007). Without adequate support, these impacts of the violence have the potential to escalate and cause further distress for the children and challenges later on in life (Taylor, 2019; Vu et al., 2016). In addition to psychoeducation on these issues, the program may benefit from linkages with a clinical child psychologist with experience in DV to whom families could be referred. This could ensure children were being diagnosed accurately and early, and receiving the support they need.

Overall, men had mixed views on the impact of the program for themselves. The most hopeful outcome among the men was increased insight and understanding of what constituted harmful behavior. They identified the ability to talk about it with their family, ex/partners and children, and some developed skills to manage their response to triggers that stimulated their abusive behavior. Evaluations of other family- and parenting-centered programs have achieved similar results; one triangulated analysis of Caring Dads programs across Melbourne, Australia, showed that the programs positively impacted men's capacity to manage their over-reactive responses within their partner and parenting relationships (Diemer et al., 2020). However, the Caring Dads programs did appear to have greater success in drawing on the parent-child relationship as a motivator for change. Again, adding more formalized components surrounding the impacts of DV on children, building parenting skills, and centering children's experiences is something that the KST program could look to focus on in any future iterations.

A further positive finding was that while some men remained resistant to taking responsibility for the abuse or spoke about the program negatively, all were engaged and intending to continue. The choice to continue provides opportunity for on-going and potential benefits. This is a notable achievement, as interventions for men/fathers are often characterized by high attrition rates, regardless of whether attendance is voluntary or court mandated (Daly & Pelowski, 2000; Fitz-Gibbon et al., 2024; Olver et al., 2011).

The format of KST support was one of the key motivators for the men. All men had positive regard for the KST practitioners, and combined with the individual one-to-one format, felt comfortable speaking and in general, felt heard. These elements were important to the men in terms of their on-going engagement. Positive feedback from both men and women in the program, about the ability of KST practitioners to connect with the men, while retaining a practice of holding them accountable for their behavior suggested that the program was not collusive—a criticism of some other programs (Costello, 2006; Reimer, 2020). It also suggests that unlike some previous “all of family” interventions (Stanley & Humphreys, 2017), practitioners held the appropriate skills, experience, and confidence to work with men who use violence in an “all of family” context. Moreover, the strength of rapport between men and their practitioners may work to overcome demotivating feelings of shame among the men, thereby minimizing risk of disengagement (Fitz-Gibbon et al., 2024). For those who do disengage, a formal process for following-up with clients may actively encourage men to attend further sessions.

It is also important to highlight that some men who were engaged in the program, especially those who were in the early stages of attendance, sometimes expressed blame towards their (ex) partners for “driving” them to use abusive behaviors. At the time of interview, the men had been in the program for a minimum of 20 weeks. While this illustrates a very short timeframe for shifting knowledge, awareness, and thought processes, 20 weeks is the standard length of a men's behavior change program in Australia (No To Violence [NTV], 2024). The fact that some men continued to blame their (ex) partners for their own abusive behavior reinforces the need for on-going and extended programs to support long-term attitudinal and behavioral shifts, as KST was designed to offer.

Some women also reported that though they initially felt safer around the ex/partner when first becoming involved in KST, they believed the change was temporary. This again reflects the need for processes that support long-term change in thinking, attitudes, and behavior. The strength of the “all of family” model is that it does allow for individuals to continue engaging independently or as a family, unlike other MBCPs where women's access to the service is often tied to men's continued engagement in the program (Smith et al., 2014). This flexibility in engagement could therefore extend further, to include the opportunity for clients to re-engage when circumstances change, or old behaviors creep back in.

The inability to shift some men's attitudes and behavior is indicative of the challenge that many behavioral change programs face. Due to the statutory nature of the referral pathways, namely child protection and courts, some participants did not attend KST voluntarily. These men may be less motivated by a genuine desire to change their behavior than to record attendance. This can result in a lack of commitment to learning and in some cases can cause men to disengage early in the program. Some motivation for change may be developed in men once they begin attending sessions, though this will not be the case for everyone. The type of motivation that perpetrators draw upon when attending behavior change programs can impact the outcomes considerably (McGinn et al., 2017). The implication for practitioners in this area may be further exploration of the men's motivations to attend in order to re-shape these motivations from “functional” (e.g., record of attendance or because their partner threatened to leave) to “existential” (e.g., because it's the right thing to do, to feel better about who they are) (McGinn et al., 2017).

One unique aspect of the KST program was that it ended up engaging families where the level of risk was higher than initially intended. Other family-based programs, such as the UK-based Steps to Safety program, screen out couples for whom couples-based work may be unsuitable (McConnell et al., 2020). This includes where parents are emotionally dysregulated, where there are severe levels of control, or where the violence is likely to escalate (McConnell et al., 2020). Due to the nature of the referral pathways into KST, the staff members instead opted to alter the program to extend supports to higher risk families. While this did not come without its challenges—and some of the shortcomings of the program may be attributed to the fact that the families were higher risk—it does show that these risks can be effectively managed in an “all of family” program.

Our findings showed that some men struggled with consistency when faced with stressors, especially in the context of AOD use, an issue beginning to be addressed in other programs for men who use violence (Kertesz et al., 2022; Stover, 2015). While the prevalence of harmful AOD use for the participants interviewed is not known, administrative data for the KST program as a whole shows that 27% of men engaged in the program identified as “being affected by a drug of addiction (including alcohol).” Providing support on AOD issues was beyond the scope of the KST program, and attendees were instead referred to external AOD support. However, in recognition that these issues may be interconnected and re-enforcing men's use of violence (Our Watch et al., 2015), future “all of family” interventions may look to integrate specialist AOD support into their programs.

Another area for potential development in “all of family” programs is the inclusion of wider family networks. The KST program focused on couples and children. However, there may be support roles that other family members can play to facilitate safety and hold men accountable for their behavior. This may be especially relevant for families living in multi-generational households. If risks related to the inclusion of other family members are managed, this could also have the added effect of preventing victim-survivor isolation and disconnection from social and cultural communities.

Lastly, an important aspect of the program to consider is effectiveness and appropriateness when working with diverse communities. Only a small number of participants in this research identified as CALD, and none as Aboriginal. However, due to analysis of administrative data, the authors do know that the majority of clients in the KST program overall are of diverse backgrounds (Spiteri-Staines et al., 2019). As highlighted in the background of this article, “all of family” programs may be particularly important to service cultural communities where there are intersecting experiences that impact women's choice or ability to leave a violent relationship. There may be issues such as insecure visa status (Humphreys & Campo, 2017) or desires to remain connected to cultural lands and communities (Meyer & Stambe, 2020; Murray, 2008). In these families, seeking support for the violence “together” may be the most appropriate, realistic, and safe option to maintain both individual as well as collective wellbeing. It was unfortunate that we were unable to interview more CALD and Aboriginal participants of the program, and this is recognized as a limitation in our ability to identify what changes could be made to better support the needs of diverse families and clients. For instance, whether there may be benefits to culturally matching clients and practitioners (Sawrikar, 2013), the need for increased training to build practitioner capacity to work across cultural and linguistic groups (Pokharel et al., 2023), or incorporating practice elements that draw from clients’ own cultural values and systems, including collaboration with community or religious leaders/Elders (Morgan et al., 2022; Vaughan et al., 2020). More research on whether “all of family” interventions can meet the needs of diverse communities specifically is therefore needed.

There are some further limitations to this study that should be addressed. Firstly, as this evaluation was commissioned in the final six months of program funding, collection of baseline interview data was not possible. Second, the program was a pilot, and the sample size was naturally limited, therefore also limiting the generalizability of the findings. Further research into similar programs, using a larger and more diverse sample across a broad range of demographics and geographic locations, would provide more detailed experiences and reveal new or confirm displayed trends. Third, data was collected in the final few months of program funding. Analysis could be further strengthened by conducting a follow-up study with the same participants to measure program impact six months to two years post-engagement. This would be beneficial to understand whether the positive changes the participants identified were maintained over a longer period.

Lastly, while some participants were able to take part in couples’ sessions, the family counseling element of the overarching program was not able to be evaluated as the participants interviewed did not meet the criteria. This aspect was designed as an optional element for situations where both partners desired family counseling, and it was deemed safe to do so. There were families in the KST program who were able to participate in family sessions that included children. Unfortunately, none of these clients consented to be interviewed and as such we do not have information on their experiences of this component. The inability to evaluate this aspect of the program is acknowledged as a limitation of the research and demonstrates the challenges of getting to this stage safely. This element would require careful monitoring if introduced into any future program.

## Conclusion

“All of family” DV programs are an alternative and under-researched method of intervention. This study provides new evidence to show that one such program, KST, can provide positive results increasing women's and children's safety and supporting men's behavioral change while families have frequent contact with one another. While improvements to the program are required—namely, increased focus on children's experiences, shifting men's motivation for attending away from relationship repair towards harm minimization, and longer or flexible engagement periods—the benefits to families were clearly articulated by the participants interviewed. Even given the short duration of the pilot program of KST, and the small sample size, this study indicates value in further exploration of all of family programs such as KST.
